# Supplementary material for: Zhang-Rice singlets state formed by two-step oxidation for triggering water oxidation under operando conditions
Source: Nat Commun. 2023 Feb 1;14:529. doi: 10.1038/s41467-023-36317-2 (PMC9892518; doi:10.1038/s41467-023-36317-2)
Supplement: Supplementary file 1 — Supplementary information [file 41467_2023_36317_MOESM1_ESM.pdf]

Supplementary Information for

Zhang-Rice singlets state formed by two-step oxidation for triggering water oxidation under *operando* conditions

Peng et al.

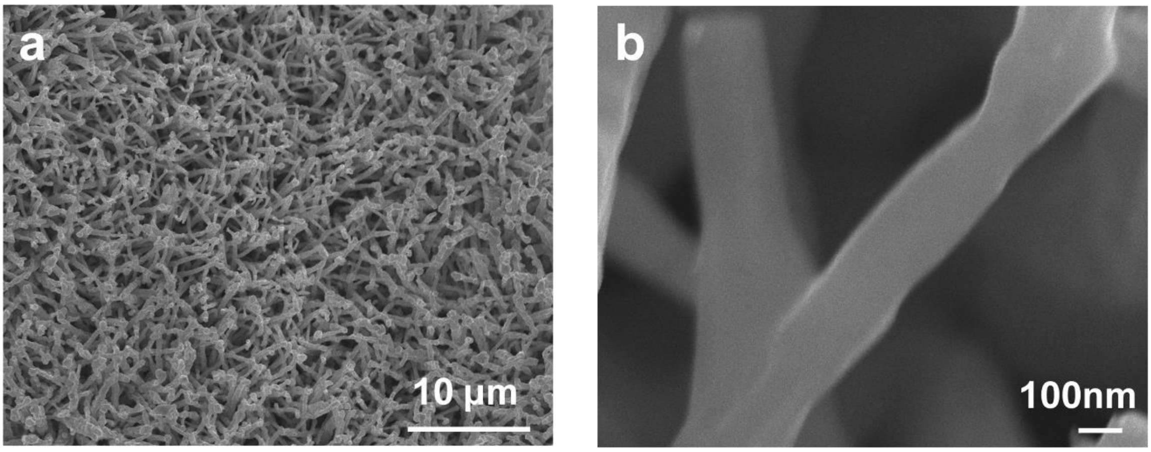

Supplementary Fig. 1 | a SEM image and **b** enlarged SEM image of H-Cu_2O catalysts.

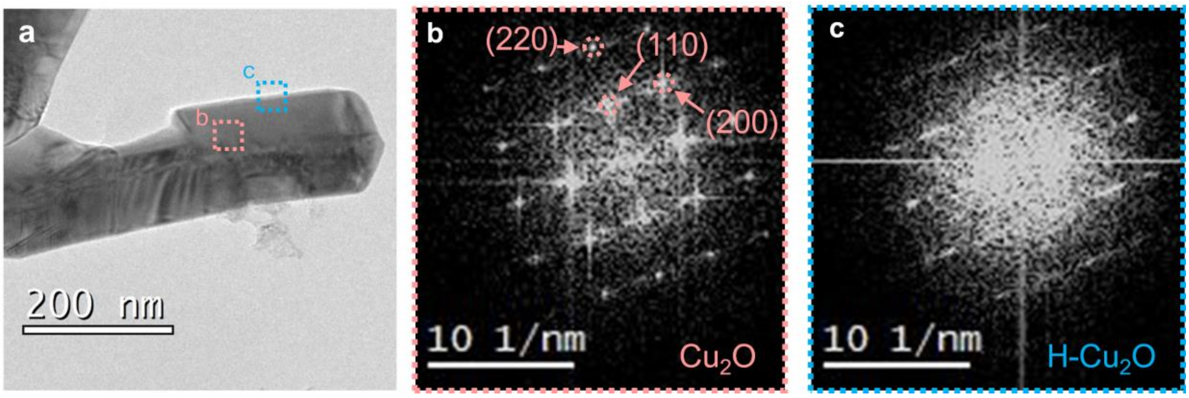

Supplementary Fig. 2 | a TEM image of H-Cu_2O catalysts. **b** corresponding FFT pattern selected from region b in **Supplementary Fig. 2a**. **c** corresponding FFT pattern selected from region c in **Supplementary Fig. 2a**.

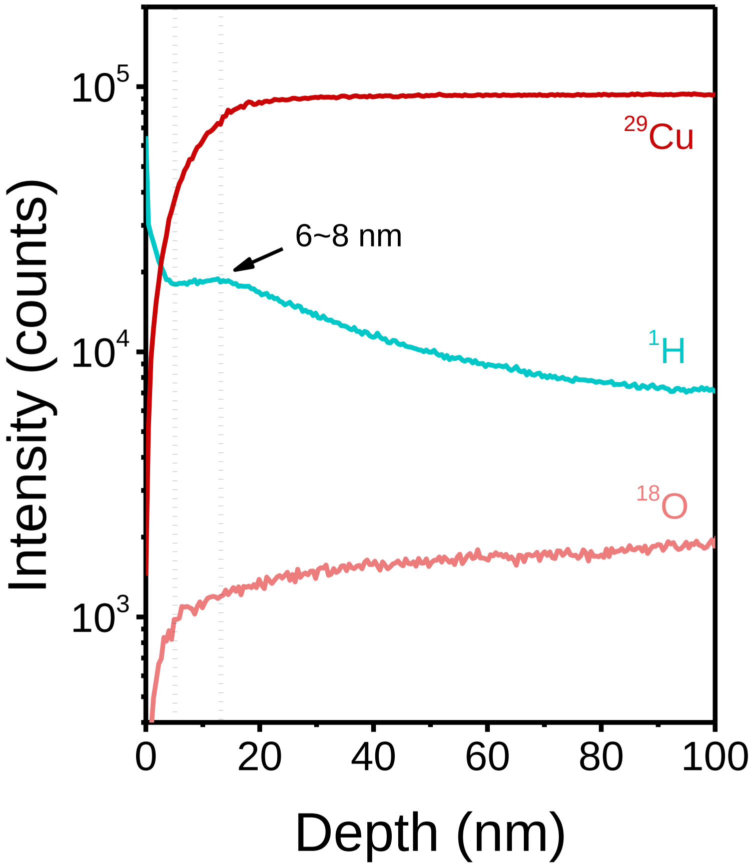

Supplementary Fig. 3 | SIMS depth profile of H-Cu_2O catalysts.

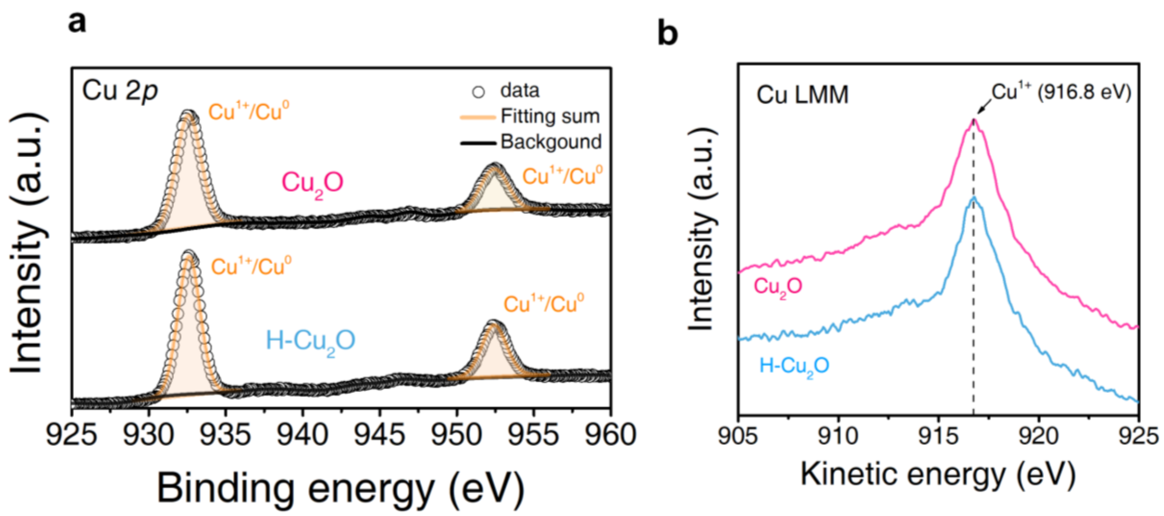

Supplementary Fig. 4 | XPS of **a** Cu 2*p* and **b** Cu LMM for the Cu_2O and H-Cu_2O catalysts.

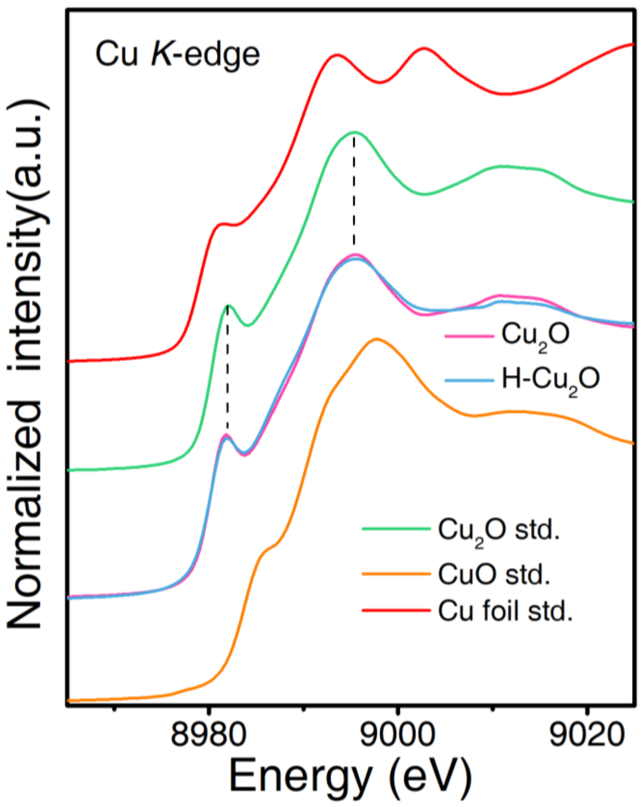

Supplementary Fig. 5 | XANES spectra of Cu *K*-edge for catalysts as prepared (std: standard).

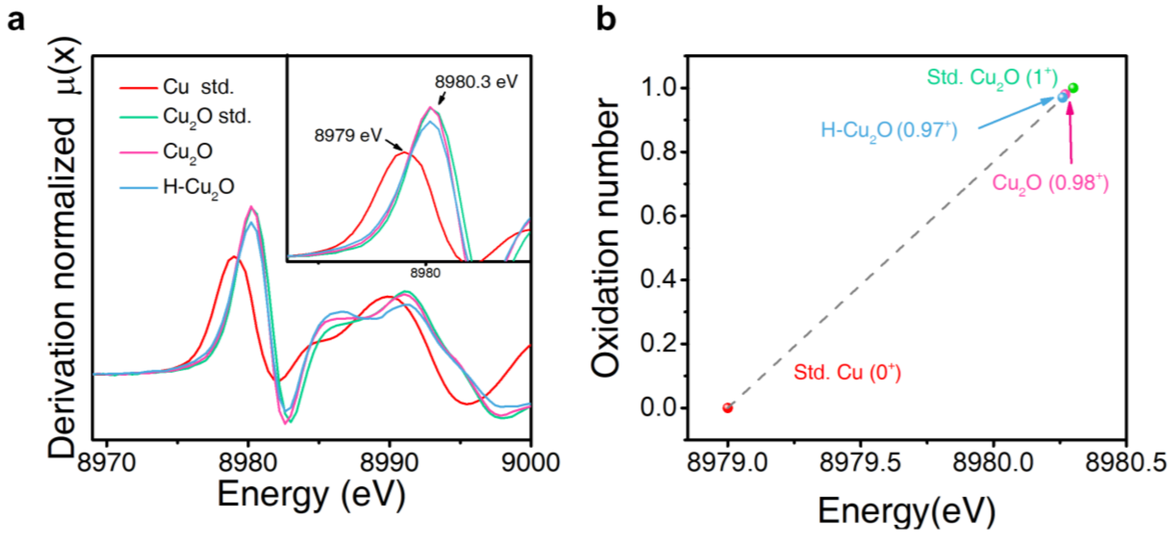

Supplementary Fig. 6 | a Corresponding first-derivative spectra at the Cu *K*-edge for catalysts as prepared; **b** reference-derived linear regression of Cu valence in catalysts as prepared (std: standard).

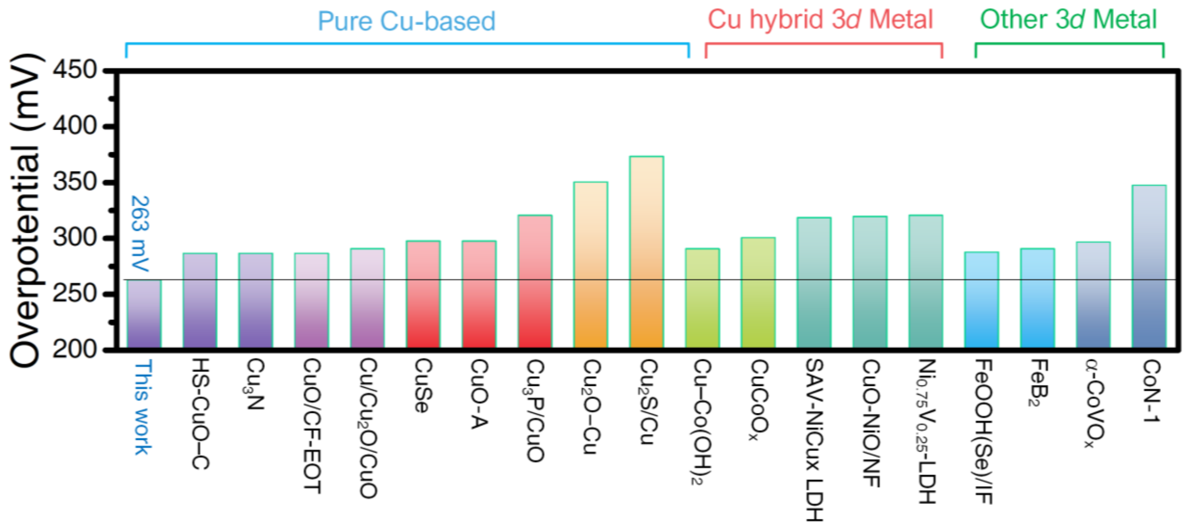

Supplementary Fig. 7 | Comparison of OER catalytic performance of H-Cu_2O catalysts with reported Cu-based catalyst and other 3*d* TM-oxide catalysts[1-18].

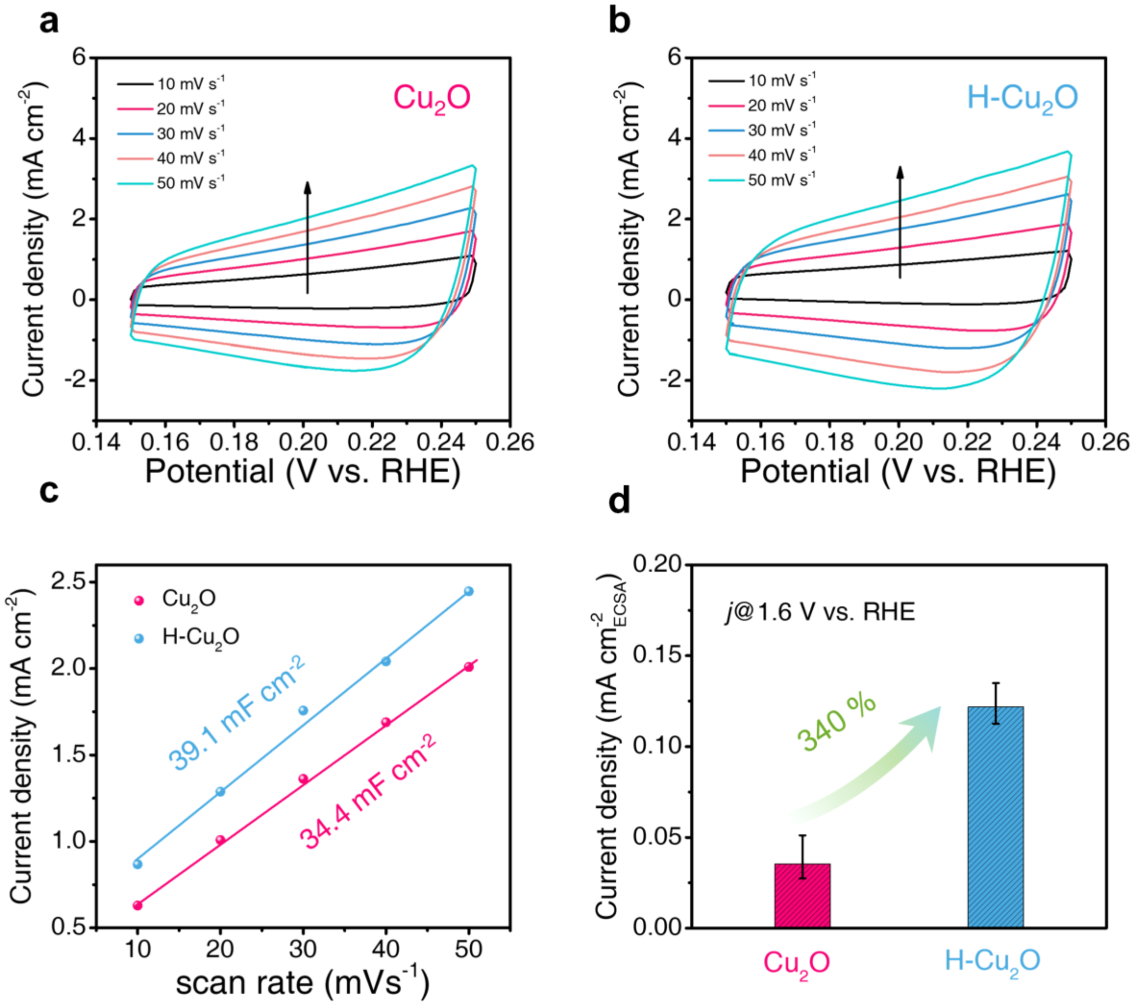

Supplementary Fig. 8 | CV curves at varied scan rates for **a** Cu_2O and **b** H-Cu_2O; **c** calculated electrochemical double-layer capacitance at varied scan rates for catalysts as prepared; **d** ECSA normalized current density recorded at 1.6 V vs. RHE for catalysts as prepared (error bars represent the standard deviation).

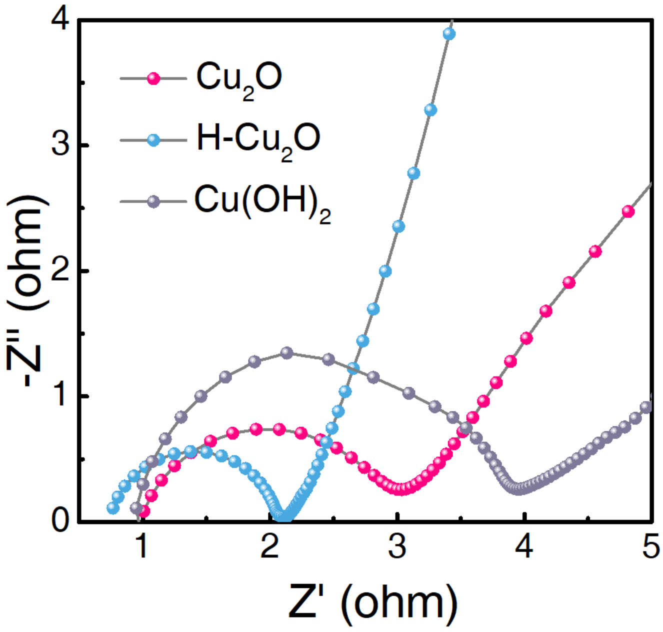

Supplementary Fig. 9 | EIS Nyquist plots recorded at 1 V vs. RHE in KOH (1 M).

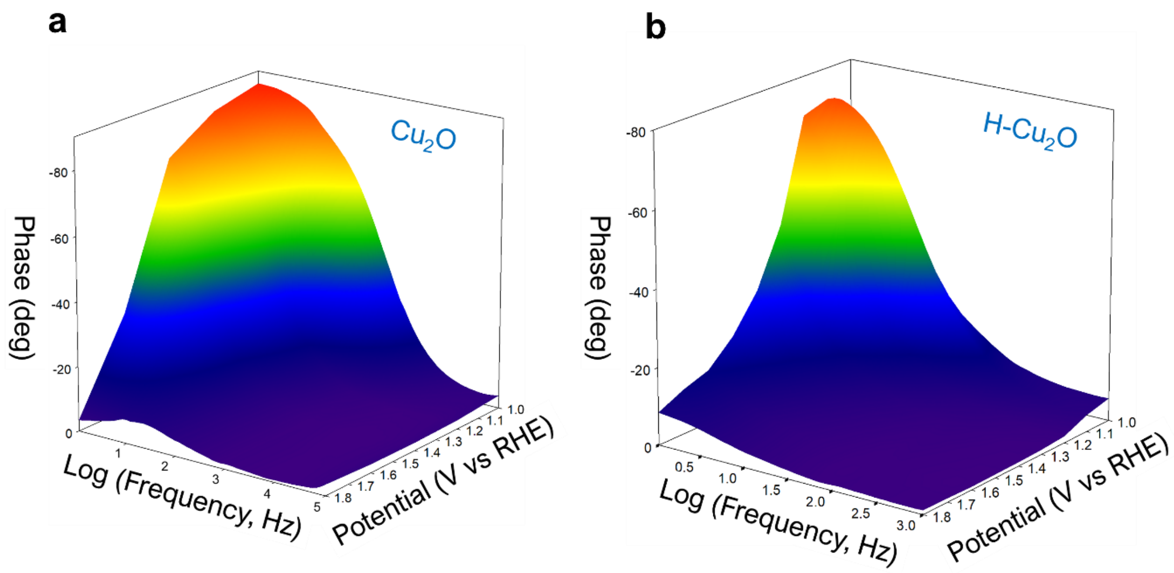

Supplementary Fig. 10 | 3D-contour Bode plots during OER for the Cu_2O and H-Cu_2O catalysts.

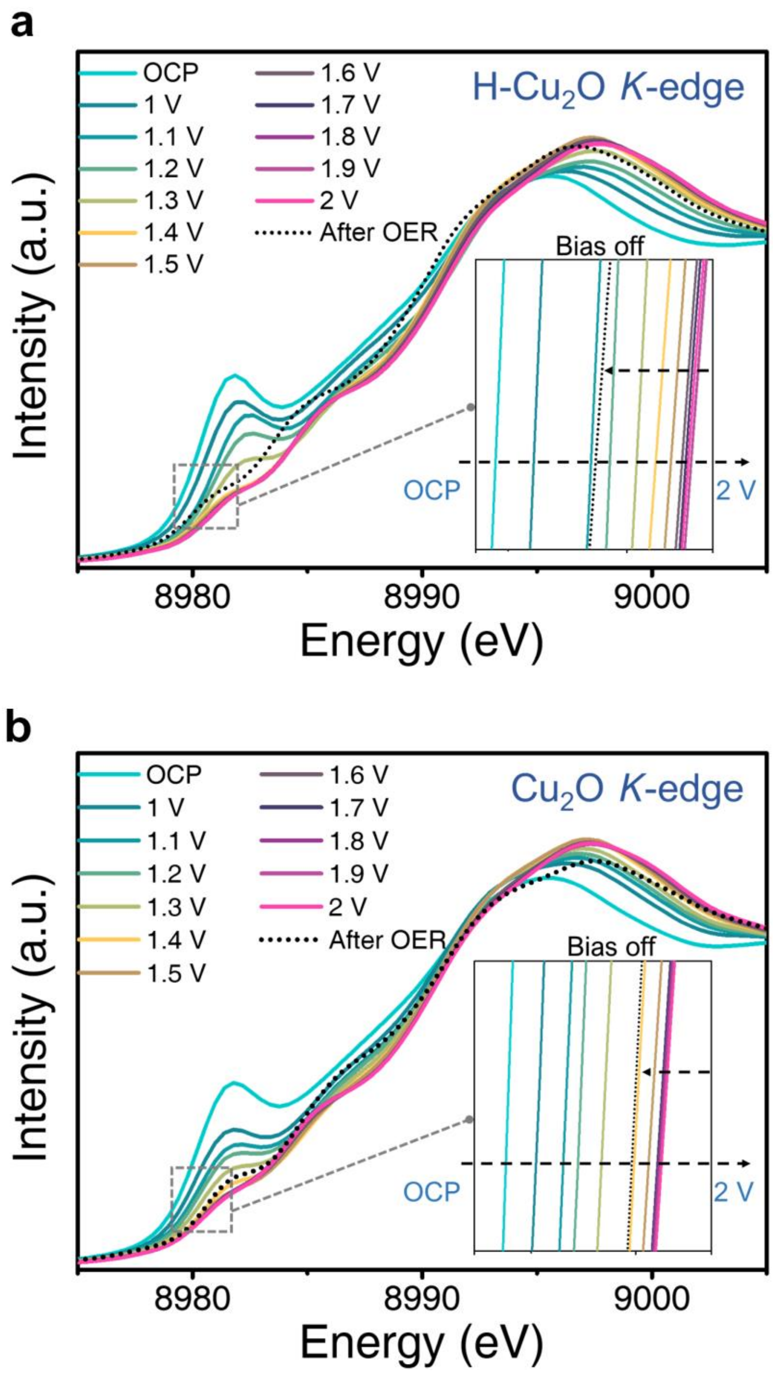

Supplementary Fig. 11 | *Operando* XANES for Cu *K*-edge of **a** H-Cu_2O and **b** pure Cu_2O.

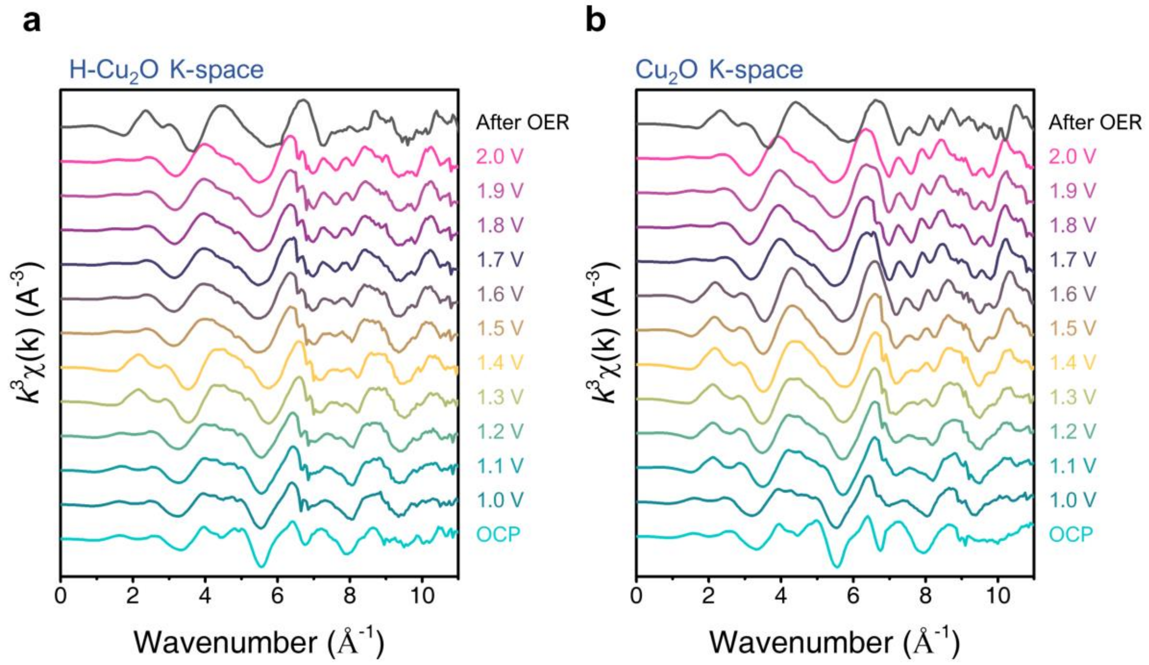

Supplementary Fig. 12 | Corresponding K-space Cu *K*-edge EXAFS spectra of **a** H-Cu_2O and **b** pure Cu_2O.

EXAFS measurements and analysis EXAFS measurements.

The X-ray absorption find structure (EXAFS) data were collected at beamline TPS BL44A in NSRRC, Taiwan. The acquired EXAFS data were processed according to standard procedures with the Athena module implemented in the IFEFFIT software packages. The k^3-weighted EXAFS spectra were obtained on subtracting the post-edge background from the overall absorption and then normalizing with respect to the edge-jump step. Subsequently, k^3-weighted χ(k) data in the k-space ranging from 3–11 $Å^{-1}$ were Fourier-transformed to real (R) space using a Hanning window (dk = 0 $Å^{-1}$) to separate the EXAFS contributions from various coordination shells.

To obtain quantitative structural parameters about Cu atoms in H-Cu_2O, we performed a parameter fit of the least-squares curve with module Artemis of IFEFFIT and USTCXAFS software packages. Effective scattering amplitudes and phase shifts for the Cu-O and Cu-Cu pairs were calculated with the code FEFF8.0 ab initio. First of all, EXAFS data at the Cu K-edge for the pristine Cu_2O counterpart were fitted. The coordination numbers of the first to third coordination shells were fixed as the nominal values; the internal atomic distances R, Debye-Waller factor σ^2, and the edge-energy shift E_0 were allowed to vary freely. The amplitude-reduction factor S_0^2 was also treated as an adjustable variable; the obtained value of 1 for pristine Cu_2O was fixed in fitting the subsequent Cu edge data for H-Cu_2O. The obtained results were all in accordance with the theoretical values, evident of the high accuracy of EXAFS in determining structural parameters. When fitting the Cu EXAFS data of H-Cu_2O, the fit was done on the k^3-weighted EXAFS function $\chi(k)$ data from 3 to 11 $Å^{-1}$ in R-range 1–3.5 Å. The coordination numbers (CN), interatomic distances (R), Debye-Waller factor (σ^2) and the edge-energy shift (ΔE_0) were allowed to vary freely. Following the above fitting strategy, we obtained satisfactory curve-fitting results as shown in Supplementary Fig. 13; the obtained parameters are listed in Supplementary Table 1-2.

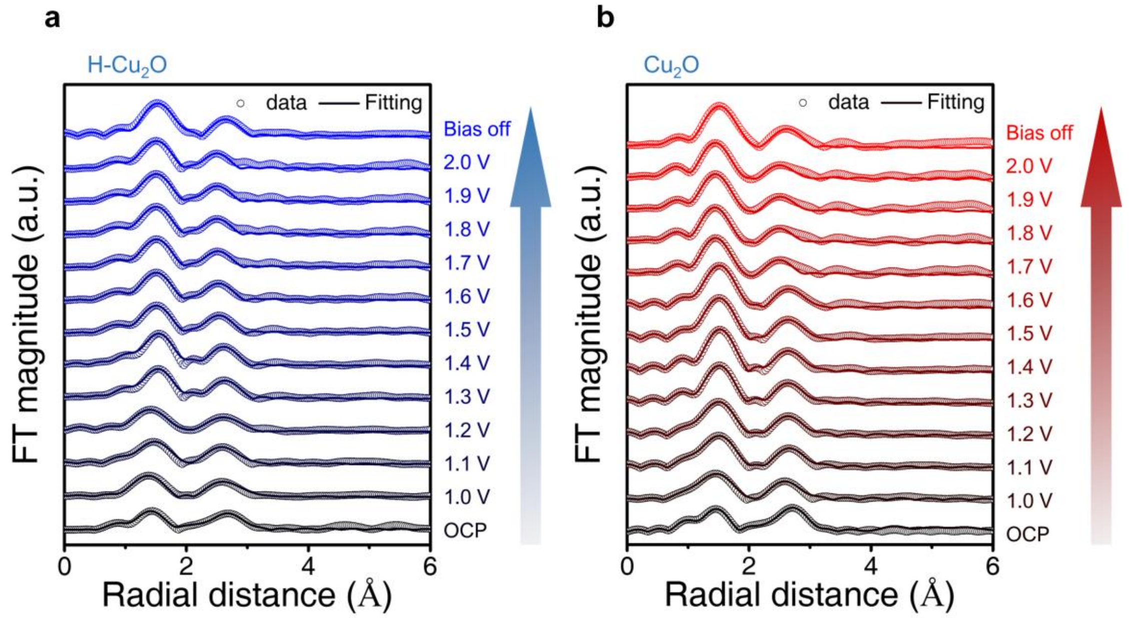

Supplementary Fig. 13 | *Operando* EXAFS spectra of **a** H-Cu_2O and **b** pure Cu_2O. The detailed results of the fit are listed in **Supplementary Table 1-2**.

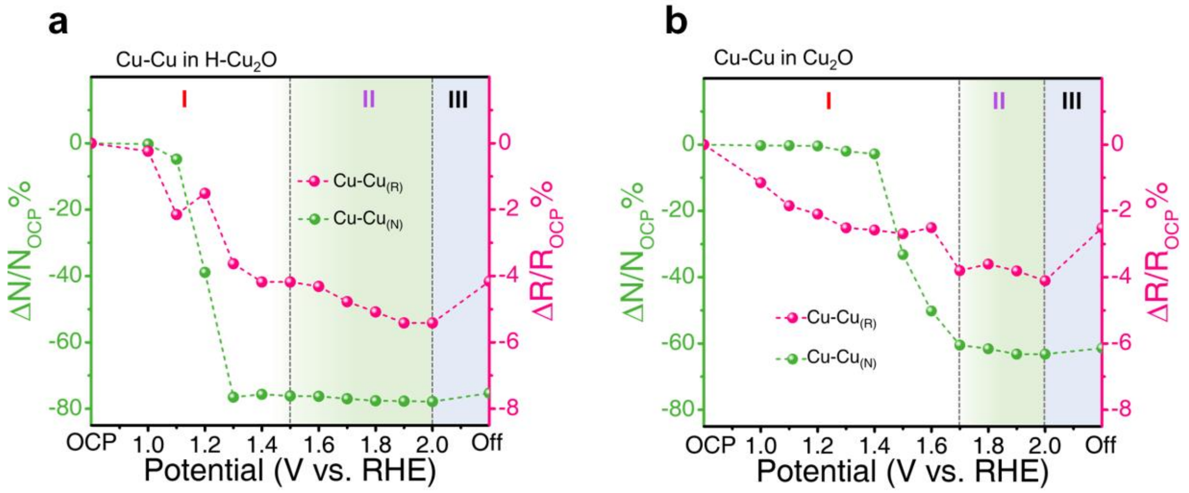

Supplementary Fig. 14 | Structural coherence changes of Cu-Cu in EXAFS coordination number (N) and bond length (R) of **a** H-Cu_2O and **b** Cu_2O under applied potential relative to the OCP state.

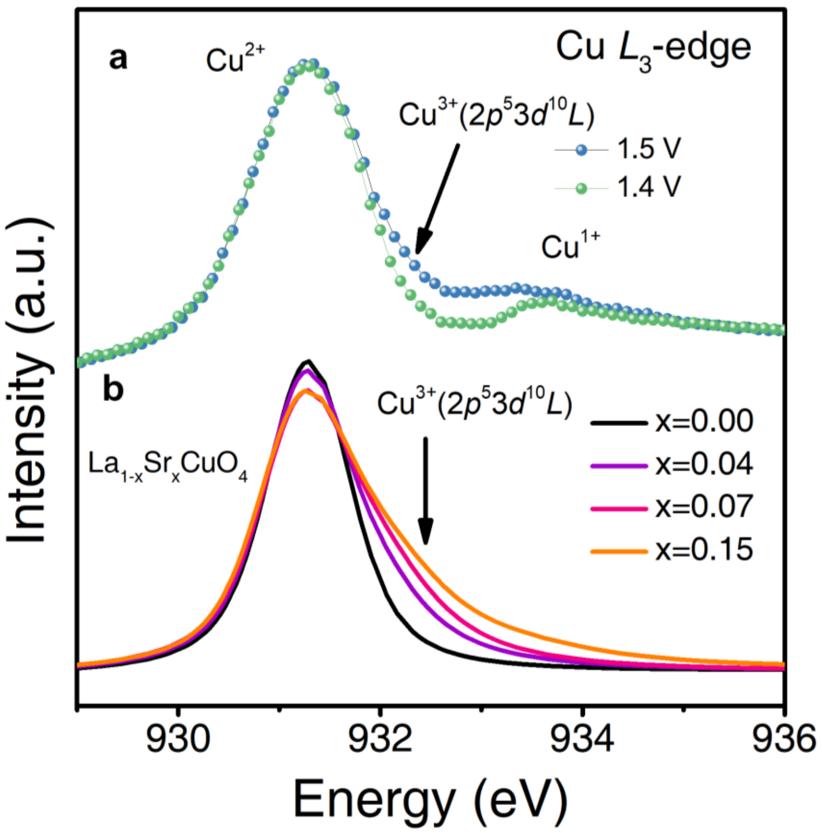

Supplementary Fig. 15 | Cu L_3-edge soft-XAS spectra. **a** H-Cu_2O at 1.4 V and 1.5 V, **b** Cu^{3+} in $La_{1-x}Sr_xCuO_4$ from reference[19].

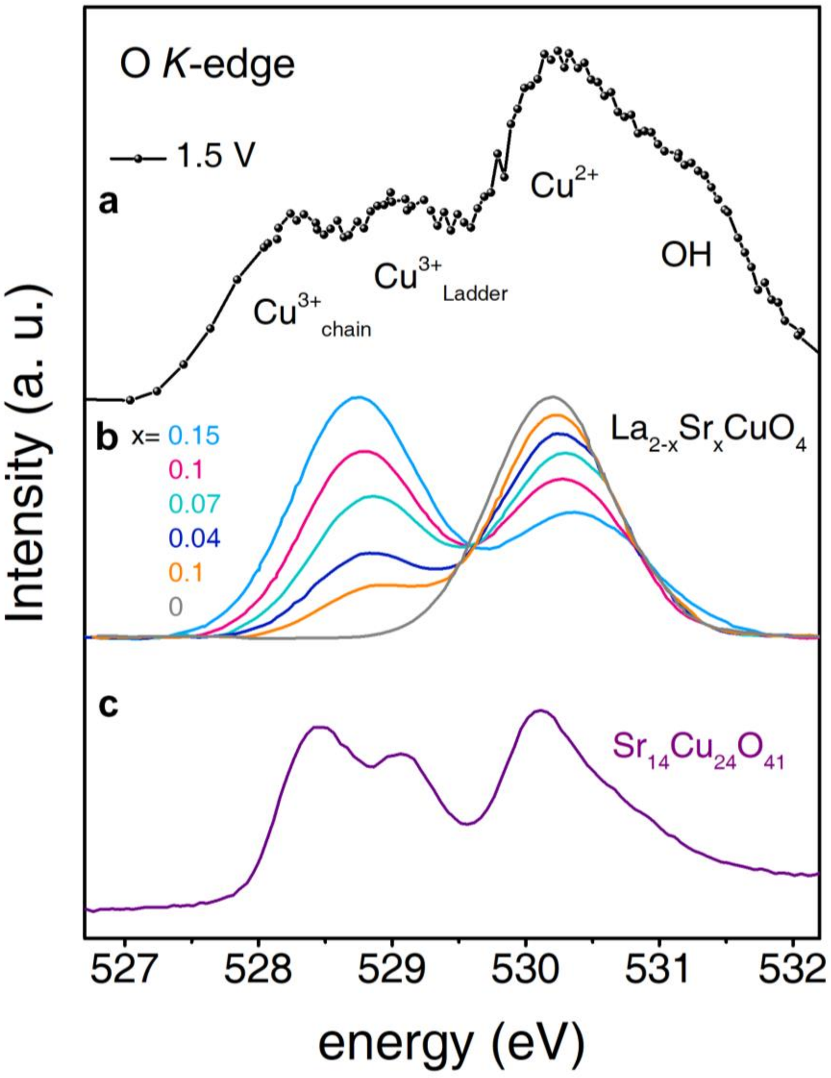

Supplementary Fig. 16 | O *K*-edge soft-XAS spectra. **a** H-Cu_2O during OER, **b** $La_{2-x}Sr_xCuO_4$ (from ref.[19]) as Cu^{3+} reference, **c** $Sr_{14}Cu_{24}O_{41}$ containing two Cu^{3+}: CuO_2 chains and leg Cu_2O_3 ladders (from ref.[20]).

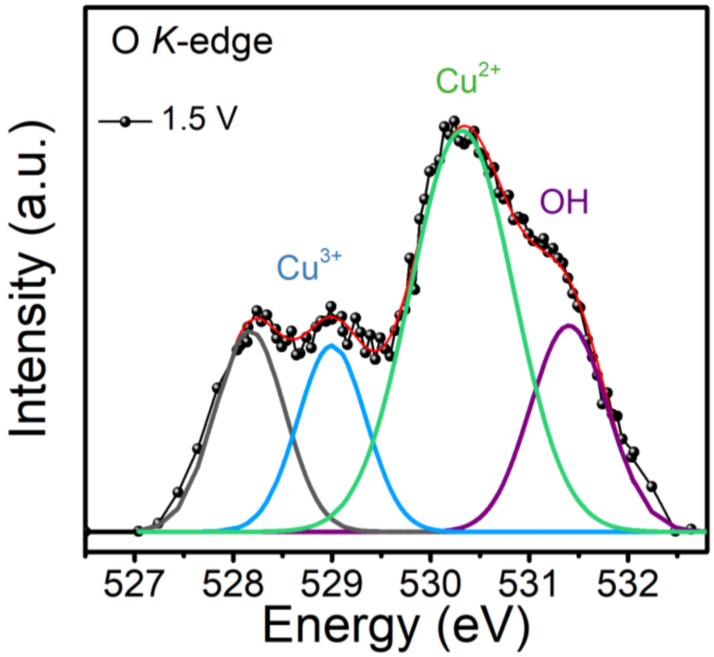

Supplementary Fig. 17 | O *K*-edge fit of H-Cu_2O during OER. The dashed blue line represents the pure corner-shared Cu-O network; the green line represents the pure edge-shared Cu-O network.

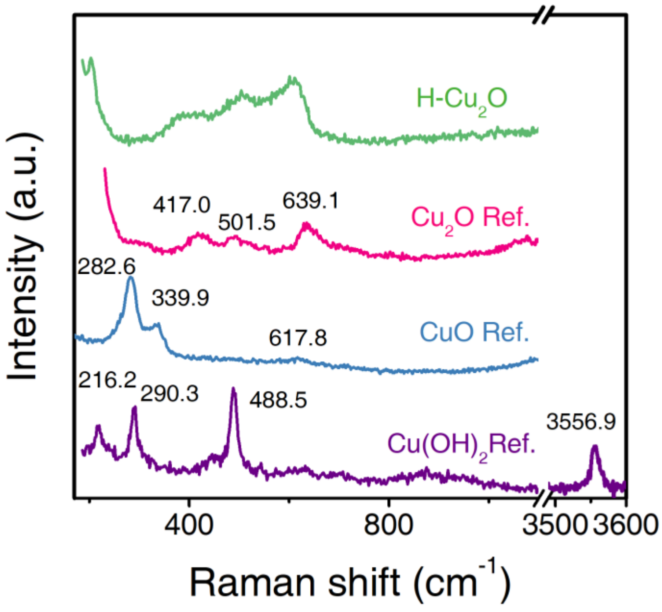

Supplementary Fig. 18 | Raman spectra of H-Cu_2O and a series of standard references (Ref.).

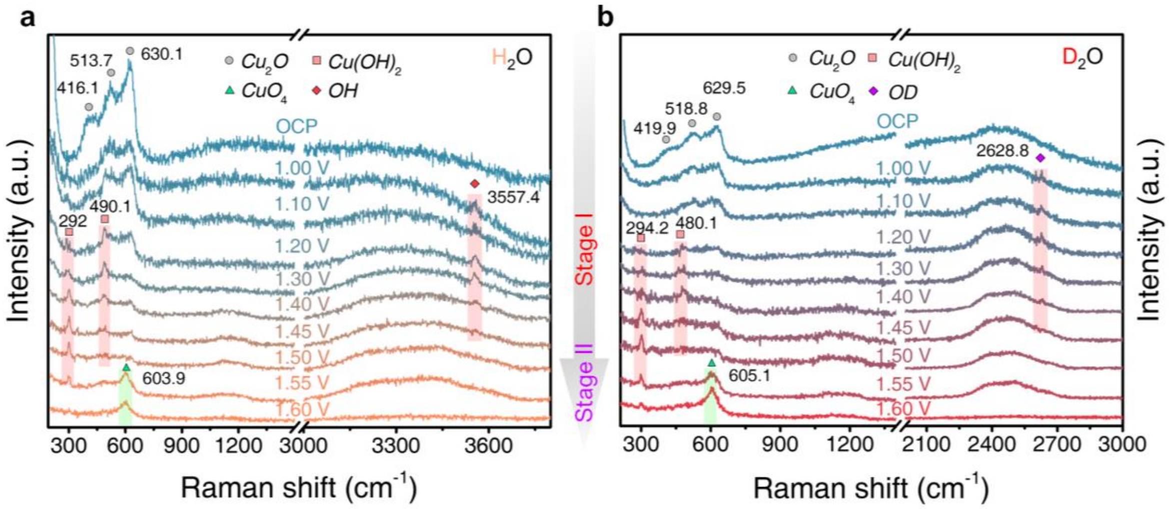

Supplementary Fig. 19 | *Operando* Raman of H-Cu_2O in **a** H_2O and **b** D_2O electrolyte.

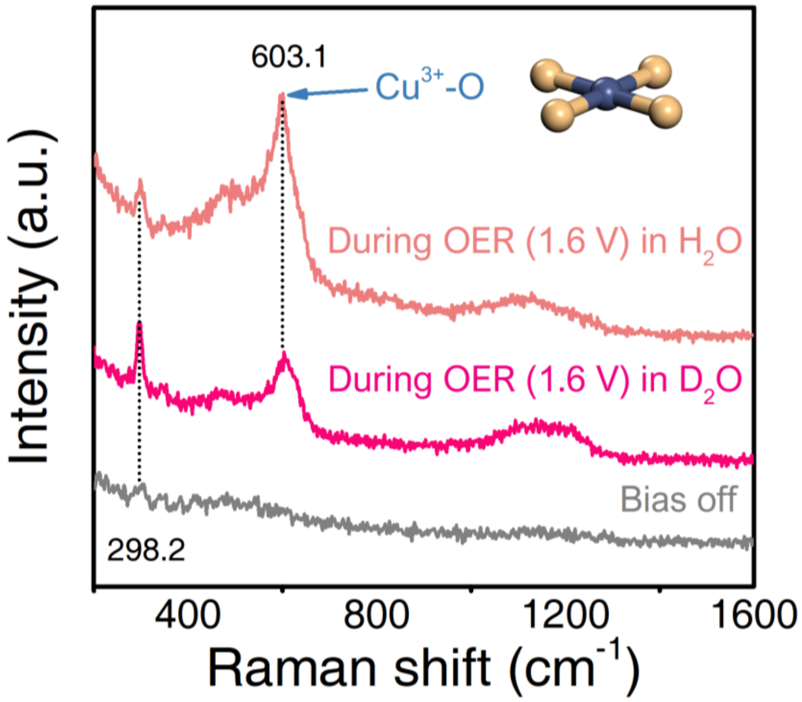

Supplementary Fig. 20 | Raman spectra of H-Cu_2O at 1.6 V during OER in H_2O and D_2O electrolyte.

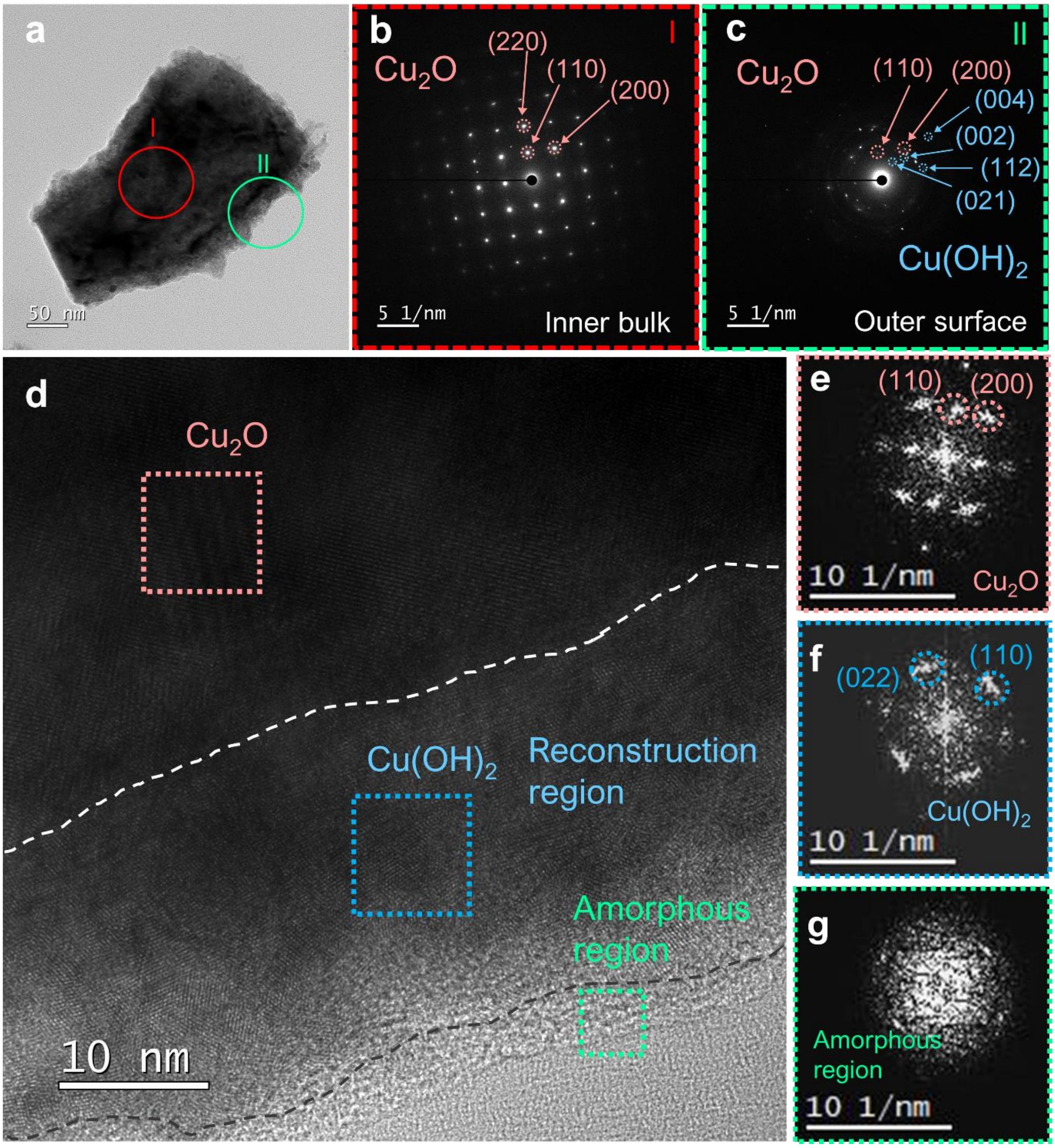

Supplementary Fig. 21 | a TEM image of H-Cu_2O after OER. SAED patterns of **b** inner bulk and **c** outer surface. **d** Cross section of H-Cu_2O after OER. The enlarged scale image and the corresponding FTT diffraction patterns of frame **e** Cu_2O core region, **f** $Cu(OH)_2$ reconstruction region and **g** amorphous region from the cross section of H-Cu_2O after OER.

Supplementary Table 1 | Summary of *operando* FT-EXAFS fit data for H-Cu_2O.

Sample	Path	CN	R /Å	σ^2/Å^2	E_0	R-factor
OCP	Cu-O	1.654	1.87251	0.0092	-5.8596	0.01609
	Cu-Cu	9.96	3.035	0.02618	-8.07442	
1 V	Cu-O	1.682	1.87125	0.00818	-5.43714	0.01317
	Cu-Cu	9.936	3.04226	0.02849	-2.54153	
1.1 V	Cu-O	1.726	1.89978	0.00897	-0.40354	0.01370
	Cu-Cu	9.48	2.96973	0.02800	-7.69081	
1.2 V	Cu-O	1.782	1.9126	0.00852	-0.39692	0.01879
	Cu-Cu	6.084	2.98916	0.02301	-6.11109	
1.3 V	Cu-O	2.146	1.93434	0.00896	9.01099	0.01829
	Cu-Cu	2.34	2.92481	0.01224	-2.89101	
1.4 V	Cu-O	2.326	1.95172	0.00893	9.552315	0.01995
	Cu-Cu	2.424	2.90807	0.01243	-0.277395	
1.5 V	Cu-O	1.954	1.93857	0.00592	-2.52865	0.01848
	Cu-Cu	2.376	2.90807	0.01233	14.39303	
1.6 V	Cu-O	1.954	1.93672	0.00572	-3.14949	0.01517
	Cu-Cu	2.364	2.90407	0.01273	14.96758	
1.7 V	Cu-O	1.996	1.93595	0.00572	-3.06587	0.01577
	Cu-Cu	2.292	2.89007	0.01293	16.61612	
1.8 V	Cu-O	1.996	1.93572	0.00572	-2.94911	0.01875
	Cu-Cu	2.232	2.8807	0.01293	17.53071	
1.9 V	Cu-O	1.998	1.93572	0.00572	-2.74521	0.01634
	Cu-Cu	2.22	2.8707	0.01313	18.33813	
2 V	Cu-O	1.996	1.93572	0.00562	-2.20372	0.01563
	Cu-Cu	2.208	2.87070	0.0132	18.01753	
After OER	Cu-O	2.170	1.94245	0.00675	10.15460	0.01943
	Cu-Cu	2.46	2.90866	0.01482	1.15165	

Supplementary Table 2 | Summary of *operando* FT-EXAFS fit data for pure Cu_2O.

Sample	Path	CN	R /Å	σ^2/Å^2	E_0	R-factor
OCP	Cu-O	2	1.85776	0.00961	-2.48564	0.01513
	Cu-Cu	12	3.03465	0.02444	-4.40275	
1 V	Cu-O	2.006	1.88502	0.0073	-3.36536	0.01576
	Cu-Cu	11.964	2.99975	0.02953	-6.04370	
1.1 V	Cu-O	2.026	1.89747	0.00525	5.68418	0.01945
	Cu-Cu	11.964	2.97862	0.0292	0.064620	
1.2 V	Cu-O	2.158	1.90011	0.00541	4.734021	0.01751
	Cu-Cu	11.952	2.97098	0.02948	0.407629	
1.3 V	Cu-O	2.35	1.91685	0.00625	6.846518	0.01893
	Cu-Cu	11.76	2.95845	0.02914	-0.036511	
1.4 V	Cu-O	2.444	1.92192	0.00581	7.768052	0.01623
	Cu-Cu	11.664	2.95650	0.02943	1.524501	
1.5 V	Cu-O	2.448	1.92592	0.00451	6.31025	0.01359
	Cu-Cu	8.016	2.95307	0.02557	1.83856	
1.6 V	Cu-O	2.482	1.92678	0.00498	8.59212	0.00740
	Cu-Cu	5.976	2.9586	0.023	2.80353	
1.7 V	Cu-O	2.204	1.91917	0.00325	-3.57565	0.01569
	Cu-Cu	4.74	2.91926	0.02102	-10.74704	
1.8V	Cu-O	2.194	1.92117	0.00343	-5.12772	0.01655
	Cu-Cu	4.608	2.92526	0.02087	-12.07090	
1.9 V	Cu-O	2.188	1.92168	0.00261	-6.57346	0.01308
	Cu-Cu	4.416	2.919	0.02	-19.75519	
2V	Cu-O	2.186	1.91921	0.00323	-5.51002	0.01356
	Cu-Cu	4.416	2.91002	0.02067	-12.63956	
After OER	Cu-O	2.478	1.92548	0.00561	10.30093	0.01025
	Cu-Cu	4.632	2.95846	0.02012	7.65782	

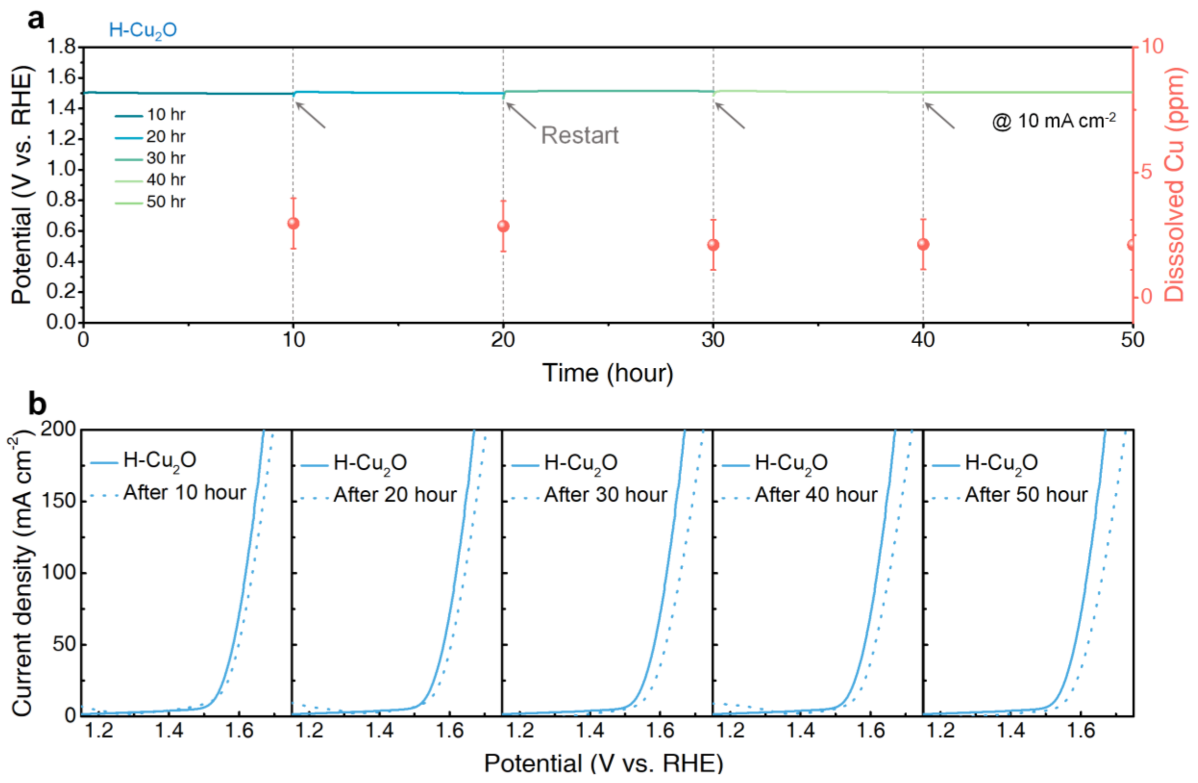

Supplementary Fig. 22 | The stability tests of catalysts. **a** Chronopotentiometry tests of H-Cu_2O and the dissolved Cu content for OER at 10 mA cm^{-2} current density after 10, 20-, 30-, 40-, and 50-hours operation. **b** LSV curves of H-Cu_2O before and after 10, 20-, 30-, 40-, and 50-hours durability test at 10 mA cm^{-2}.

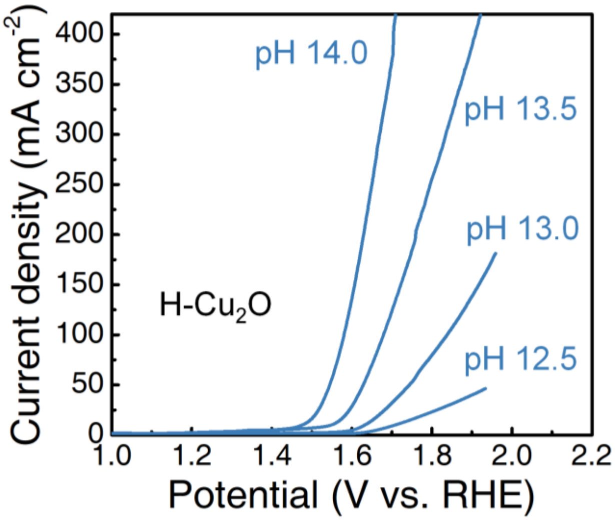

Supplementary Fig. 23 | LSV curves of H-Cu_2O recorded from pH 12.5 to pH 14.0.

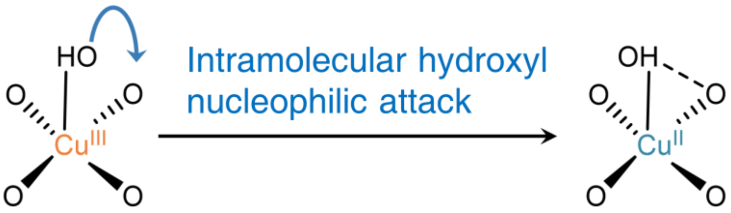

Supplementary Fig. 24 | The intramolecular hydroxyl nucleophilic attack process during OER cycle.

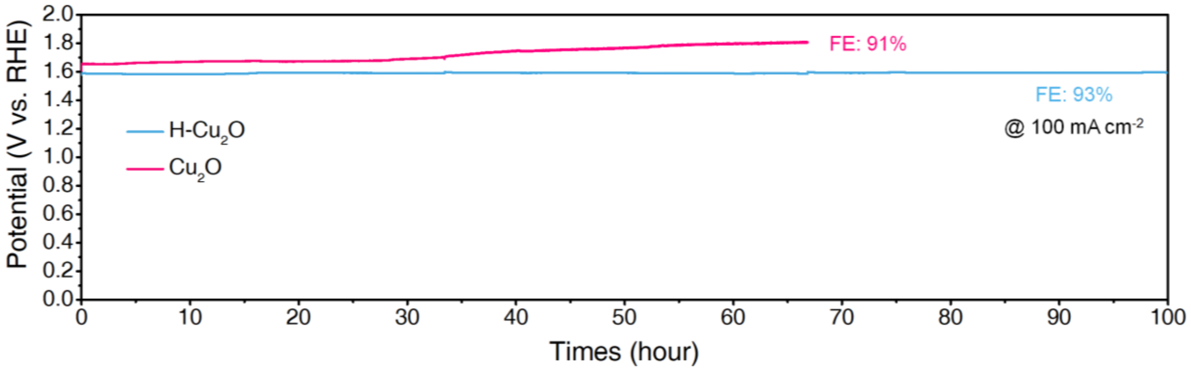

Supplementary Fig. 25 | Extended chronoamperometry measurement at current density of 100 mA cm^{-2} for 100 hours (FE: Faradic efficiency).

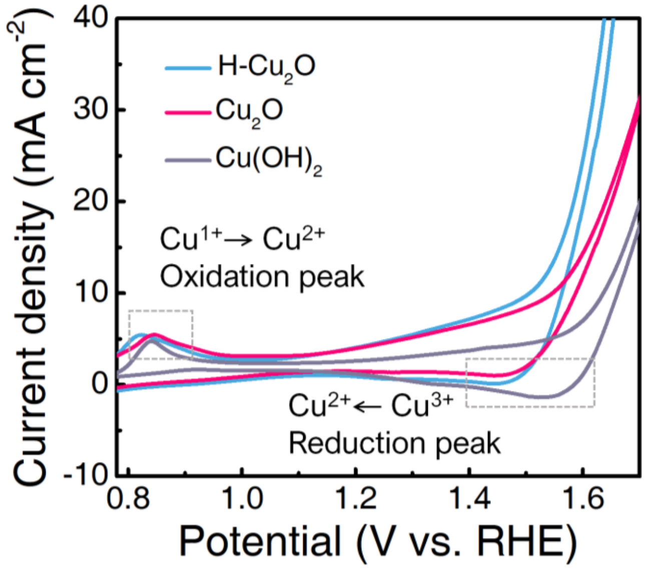

Supplementary Fig. 26 | CV curves of H-Cu_2O, Cu_2O, and $Cu(OH)_2$ recorded at scan rate of 5 mV s^{-1}.

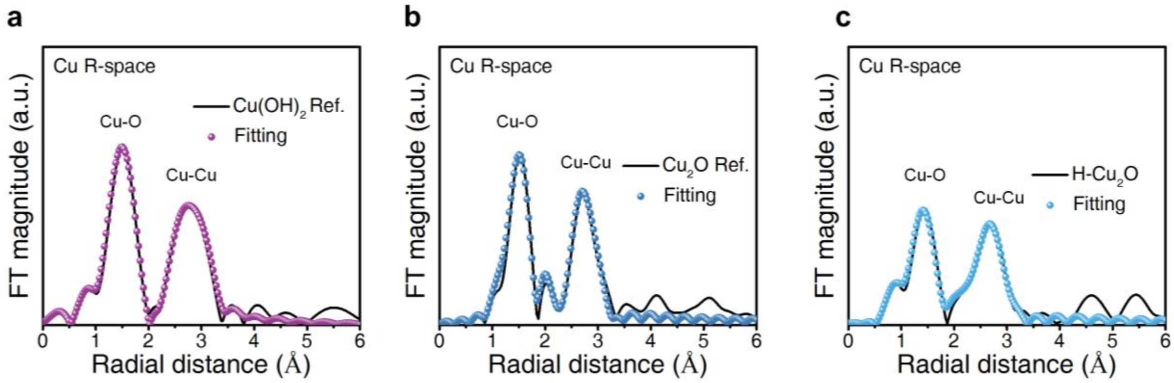

Supplementary Fig. 27 | EXAFS fitting spectra of **a** $Cu(OH)_2$ reference, **b** Cu_2O reference, and **c** H-Cu_2O (Ref.: reference). The EXAFS fitting spectra for $Cu(OH)_2$ reference, Cu_2O reference and H-Cu_2O can be found in the Supplementary dataset.

Supplementary Table 3 | EXAFS fitting results for the structural parameters around Cu atoms.

Sample	Path	CN	R /Å	σ^2/Å^2	E_0	R-factor
Cu_2O Reference	Cu-O	2	1.85207	0.00524	8.421	0.01663
	Cu-Cu	12	3.03349	0.02132	8.075	
$Cu(OH)_2$ Reference	Cu-O	4	1.95044	0.00599	-2.065	0.00729
	Cu-O	1	2.55788	0.00699	14.389	
	Cu-Cu	2.84	3.06211	0.00620	17.882	
H-Cu_2O	Cu-O	1.637	1.86251	0.00845	-3.8578	0.01749
	Cu-Cu	9.74	3.04512	0.02418	-6.07742	

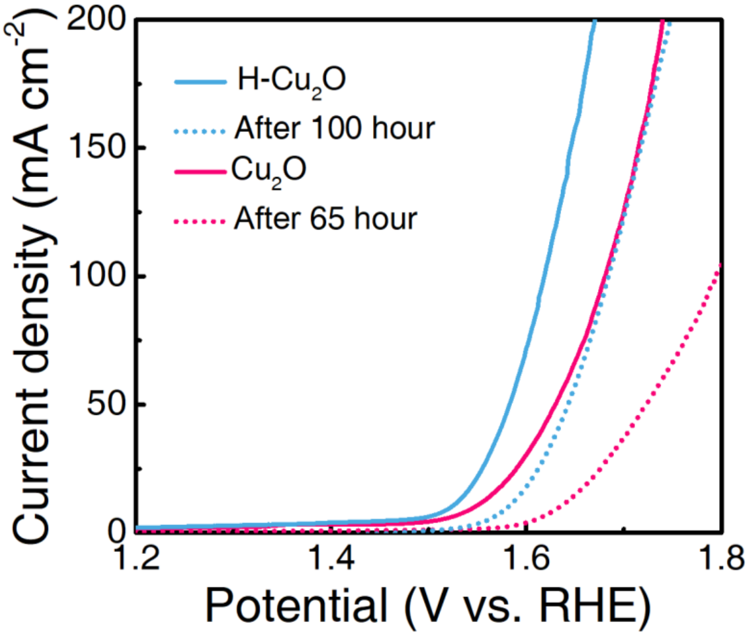

Supplementary Fig. 28 | Polarization curves of H-Cu_2O and Cu_2O were recorded before and after durability test at 100 mA cm^{-2}.

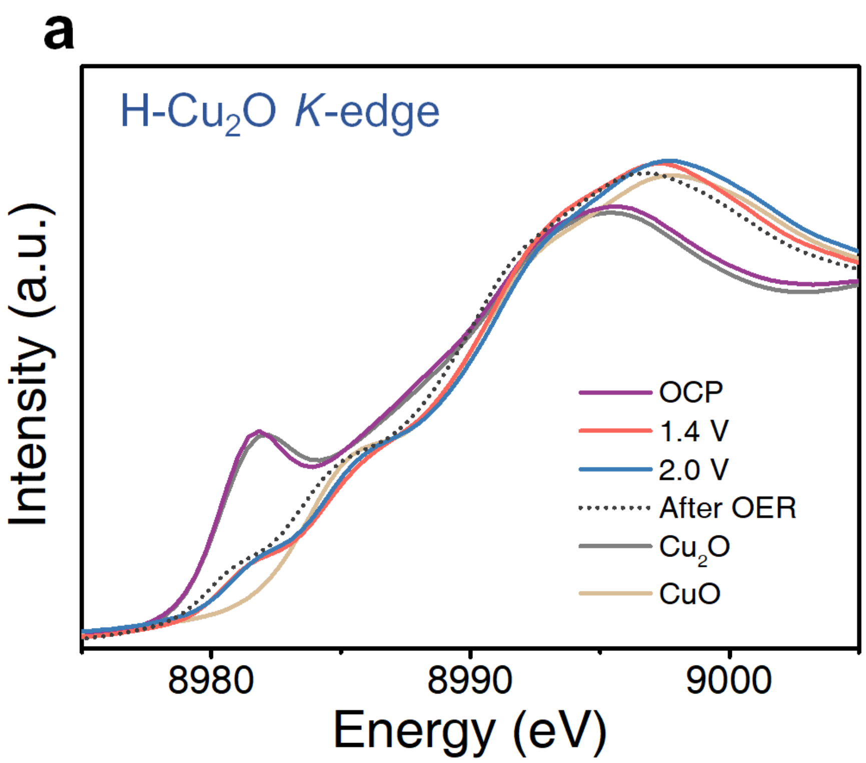

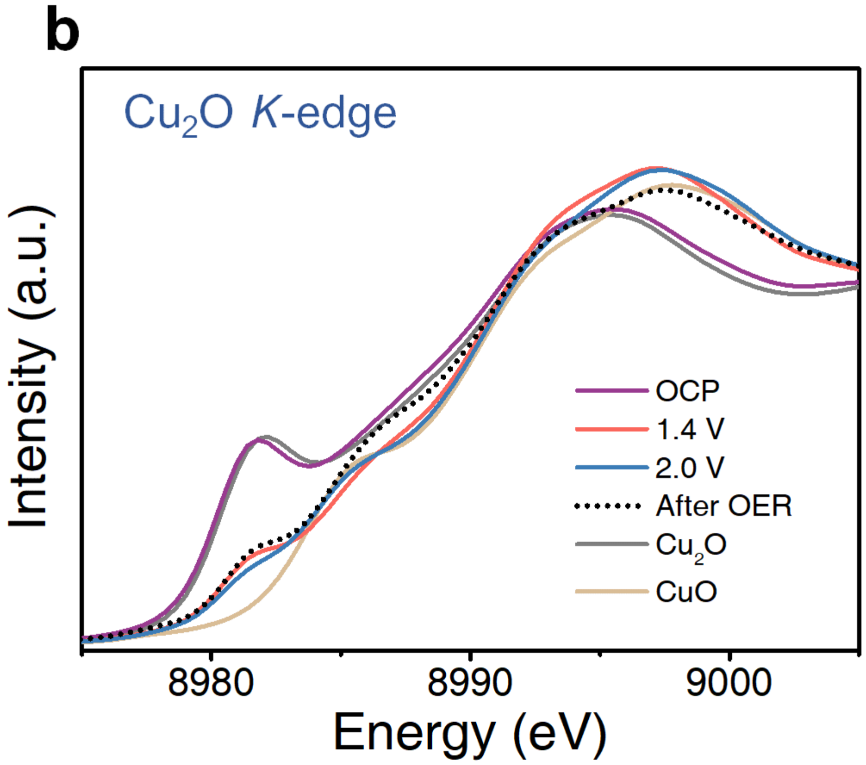

Supplementary Fig. 29 | Selective *operando* XANES for Cu *K*-edge of **a** H-Cu_2O and **b** pure Cu_2O together with the reference samples.

Reference

1. Huan, T. N. et al. A dendritic nanostructured copper oxide electrocatalyst for the oxygen evolution reaction. *Angew. Chem., Int. Ed.* **56**, 4792-4796 (2017).
2. Zhang, B. et al. Nanostructured CuO/C hollow shell@3D copper dendrites as a highly efficient electrocatalyst for oxygen evolution reaction. *ACS Appl. Mater. Interfaces.* **10**, 23807-23812 (2018).
3. Chakraborty, B. et al. Crystalline copper selenide as a reliable non-noble electro(pre) catalyst for overall water splitting. *ChemSusChem*. **13**, 3222-3229. (2020).
4. Han, A. et al. Crystalline copper phosphide nanosheets as an efficient janus catalyst for overall water splitting. *ACS Appl. Mater. Interfaces*. **9**, 2240-2248 (2017).
5. Panda, C. et al. In situ formation of nanostructured core–shell Cu_3N–CuO to promote alkaline water electrolysis. *ACS Energy Lett*. **4**, 747-754 (2019).
6. Xu, H. et al. Cu_2O–Cu hybrid foams as high-performance electrocatalysts for oxygen evolution reaction in alkaline media. *ACS Catal.* **7**, 986-991 (2017).
7. Zuo, Y. et al. In situ electrochemical oxidation of Cu_2S into CuO nanowires as a durable and efficient electrocatalyst for oxygen evolution reaction. *Chem. Mater*. **31**, 7732-7743 (2019).
8. Wang, X. et al. Copper selenide–derived copper oxide nanoplates as a durable and efficient electrocatalyst for oxygen evolution reaction. *Energy Technol*. **8**, 2000142 (2020).
9. Sun, J. et al. Cuprous sulfide derived CuO nanowires as effective electrocatalyst for oxygen evolution. *Appl. Surf. Sci*. **547**, 149235 (2021).
10. Chen, L. et al. Facile synthesis of Cu doped cobalt hydroxide (Cu–Co $(OH)_2$) nano-sheets for efficient electrocatalytic oxygen evolution. *J. Mater. Chem. A.* **5**, 22568-22575 (2017).
11. Kuang, M. et al. $CuCoO_x$/FeOOH core–shell nanowires as an efficient bifunctional oxygen evolution and reduction catalyst. *ACS Energy Lett.* **2**, 2498-2505 (2017).
12. Xie, Y.-S. et al. Dispersing transition metal vacancies in layered double hydroxides by ionic reductive complexation extraction for efficient water oxidation. *Chem. Sci*. **10**, 8354-8359 (2019).
13. Li, C. et al. Self-assembled Cu-Ni bimetal oxide 3D in-plane epitaxial structures for highly efficient oxygen evolution reaction. *Appl. Catal. B: Environ.* **244**, 56-62 (2019).
14. Fan, K. et al. Nickel–vanadium monolayer double hydroxide for efficient electrochemical water oxidation. *Nat. Commun*. **7**, 1-9 (2016).
15. Niu, S. et al. Se-doping activates FeOOH for cost-effective and efficient electrochemical water oxidation. *J. Am. Chem. Soc*. **141**, 7005-7013 (2019).
16. Li, H. et al. Earth-abundant iron diboride (FeB_2) nanoparticles as highly active bifunctional electrocatalysts for overall water splitting. *Adv. Energy Mater.* **7**, 1700513 (2017).
17. Liardet, L. et al. Amorphous cobalt vanadium oxide as a highly active electrocatalyst for oxygen evolution. *ACS Catal.* **8**, 644-650 (2018).

18. Zhang, Y. et al. Rapid synthesis of cobalt nitride nanowires: highly efficient and low-cost catalysts for oxygen evolution. *Angew. Chem., Int. Ed.* **128**, 8812-8816 (2016).
19. Chen, C. et al. Electronic states in $La_{2-x}Sr_xCuO_{4+\delta}$ probed by soft-x-ray absorption. *Phys. Rev. Lett.* **66**, 104 (1991).
20. Huang, M.-J. et al. Determination of hole distribution in $Sr_{14-x}Ca_xCu_{24}O_{41}$ using soft x-ray absorption spectroscopy at the Cu L_3 edge. *Phys. Rev. B.* **88**, 014520 (2013).
